# Illinois State Medical Society

**Published:** 1850-07

**Authors:** 


### ARTICLE II.

### ILLINOIS STATE MEDICAL SOCIETY.

Agreeably to notice calling a State Convention for the purpose, physicians from different parts of the State met at Springfield, on the 4th of June, 1850, and organized a State Medical Society, by adopting a Constitution and a Code of Ethics on the model of those of the American Medical Association.

The officers elected for the ensuing year are as follows :—

*President,*—Dr. Wm. B. Herrick, of Chicago.

*Vice-Presidents,*—

Dr. Rodolphus Rouse, of Peoria.

Dr. A. G. Henry, of Springfield.

*Secretaries,*—

Dr. Edwin G. Meek, of Chicago.

Dr. S. A. Paddock, of Princeton.

*Treasurer,*—Dr. J. A. Halderman, of Carlinville.

The following are the Standing Committees :—

*On Practical Medicine,* Drs. Samuel Thompson, of Albion, A. G. Henry, of Springfield, and Daniel Stahl, of Quincy.

*On Surgery*, Drs. D. Brainard, of Chicago, J. A. Halderman, of Carlinville, and E. S. Cooper, of Peoria.

*On Obstetrics*, Drs. John Evans, of Chicago, R. Rouse, of Peoria, and M. Helm, of Springfield.

*On Drugs and Medicines*, Drs. J. V. Z. Blaney, of Chicago, E. R. Roe, of Jacksonville, and B. K. Hart, of Alton.

*On Publication*, Drs. E. G. Meek and S. A. Paddock, Secretaries, and J. A. Haldeman, Treasurer, *ex-officio*.

Committee of Arrangements, Drs. McNeil, Arnold, and Cooper, of Peoria, the place appointed for the next meeting of the Society.

Drs. Boal, of Lacon, A. E. Ames, of Roscoe, and McNeil, of Peoria, were appointed a committee to draft a memorial to the State Legislature, asking a law providing for the registration of Births, Deaths, and Marriages, and to circulate it among the people for signature, to be presented at the next session.

A resolution was passed, directing members to discountenance the sale of secret and patent nostrums, on the part of druggists, and to patronize, as far as practicable, those only who abstain from it.

The organization of local medical societies was recommended, and they invited to send delegates to the meetings of the State Society.

The Committee on Practical Medicine were requested to procure the keeping of meteorological records in as many places throughout the State as circumstances will admit.

Dr. E. R. Roe, of Jacksonville, was appointed to deliver a public address at the next meeting of the Society, with Dr. Davis, of Chicago, as alternate.

With much harmony of feeling, and strong confidence in the success of the Society, it adjourned to meet in Peoria on the first Tuesday in June next.

We are sorry our limits will not allow a fuller abstract of its proceedings. E.

